# Microbiome analysis and fecal microbiota transfer in pediatric gastroenterology — a structured online survey in German-speaking countries

**DOI:** 10.1007/s00384-023-04351-7

**Published:** 2023-03-03

**Authors:** Alicia Brenig, Ilse Broekaert, Patrick Gerner, Carsten Posovszky, Christoph Hünseler, Alexander Joachim

**Affiliations:** 1 Department of Pediatrics, Faculty of Medicine and University Hospital Cologne, University of Cologne, Cologne, Germany; 2 Department of Pediatrics and Adolescent Medicine, Medical Center and Faculty of Medicine, University of Freiburg, Freiburg, Germany; 3 Department of Pediatrics and Adolescent Medicine, University Ulm Medical Centre, Ulm, Germany

**Keywords:** Microbiota, Composition, Survey, FMT, Dysbiosis

## Abstract

**Purpose:**

To assess the current attitude and the status quo towards the use of microbiome analysis and fecal microbiota transfer (FMT) in pediatric patients in German-speaking pediatric gastroenterology centers.

**Methods:**

A structured online survey among all certified facilities of the German-speaking society of pediatric gastroenterology and nutrition (GPGE) was conducted from November 01, 2020, until March 30, 2021.

**Results:**

A total of 71 centers were included in the analysis. Twenty-two centers (31.0%) use diagnostic microbiome analysis, but only a few perform analysis frequently (2; 2.8%) or regularly (1; 1.4%). Eleven centers (15.5%) have performed FMT as a therapeutic approach. Most of these centers use individual in-house donor screening programs (61.5%). One-third (33.8%) of centers rate the therapeutic impact of FMT as high or moderate. More than two-thirds (69.0%) of all participants are willing to participate in studies assessing the therapeutic effect of FMT.

**Conclusions:**

Guidelines for microbiome analyses and FMT in pediatric patients and clinical studies investigating their benefits are absolutely necessary to improve the patient-centered care in pediatric gastroenterology. The long-term and successful establishment of pediatric FMT centers with standardized procedures for patient selection, donor screening, application route, volume, and frequency of use is highly required to obtain a safe therapy.

**Supplementary Information:**

The online version contains supplementary material available at 10.1007/s00384-023-04351-7.

## Introduction

The human microbiome, as the collective microbiota colonizing surfaces outside and inside the human body, shows individual composition depending on multiple factors. Intact microbiota composition (eubiosis) is crucial for health whereas disturbance (dysbiosis) is associated with disease [1]. Dysbiosis is associated with a variety of diseases, such as recurrent Clostridioides difficile infection (rCDI) [2], inflammatory bowel disease (IBD) [3, 4], irritable bowel syndrome (IBS) [5], autism spectrum disorders [6], and obesity [7]. The analysis of fecal microbiota composition is of emerging scientific interest. With new sequencing techniques, microbiome analysis is accessible to a wide range of scientists and clinicians [8]. Interestingly, while the scientific approach has become more advanced, the implications for clinicians remain mostly unclear [8]. The analysis of microbiota composition is mostly not covered by medical insurance; the costs have to be covered by the patients, other hospital budgets, or through clinical trials. Modifying the gut microbiome with nutrition and medication is an essential therapeutic approach in pediatric gastroenterology [1]. Fecal microbiota transfer (FMT) is a therapeutic option to treat dysbiosis. Concerning rCDI, FMT is implemented into official treatment guidelines [2, 9]. In pediatric gastroenterology, FMT is used in individual cases. Centers performing FMT seem to differ in terms of application routes, volumes, frequencies, donors, and screening methods [10, 11]. As there is no registry for FMT, data must be assessed mainly through surveys and case reports [10]. Available data from previous surveys show that FMT is already widely used in adult gastroenterology and has high safety standards [10]. There is a lack of information on pediatric centers. We describe the status quo in German-speaking centers for pediatric gastroenterology regarding the analysis of fecal microbiota composition and therapeutic transfer of fecal microbiota

## Methods

We conducted a structured online survey among German-speaking centers for pediatric gastroenterology to evaluate the performance and their assessment of the relevance of microbiome analysis and fecal microbiota transfer (FMT) in pediatric patients. The questionnaire was developed by the team of pediatric gastroenterology at the University Hospital Cologne and was checked and pretested by the chairs of the GPGE and interested members (5 participants for pretests). After feedback, the questionnaire was adapted and completed. After approval by the local ethics committee (reference no. 20–1383), the survey was conducted via the online tool Unipark^®^. Eligible centers were identified via the network of the GPGE. The centers were invited via email and the GPGE newsletter. Centers were asked to participate only once. The survey was only available online and was carried out anonymously. Only the location of centers was identified. The survey was scheduled and open from November 01, 2020, until March 30, 2021. The survey comprised a total of 20 questions. Both open and closed questions with the option of free text answers were used.

First, general information about the participating center was asked (questions 1 and 2, size and experience of centers), followed by questions about the analysis of microbiota composition (questions 3–6, frequency, techniques, and diagnostic value). Questions 7–19 assessed information on FMT (number of FMTs performed, indications, donor selection and screening, frequency, adverse events, and patients’ views). We also asked about willingness to transfer patients to other centers for FMT and to participate in studies evaluating the efficacy and feasibility of FMT. Finally, we asked for other therapies used to influence the composition of the microbiota (question 20).

After closing the survey, data was exported and cleaned. The remaining data sets were evaluated via Microsoft Excel, Version 16.54, and IBM SPSS Statistics, Version 27. Descriptive statistics were carried out, and corresponding graphics and charts were produced. Items were evaluated using cross tables, chi-square tests, Fisher’s tests, unpaired *t*-tests, and the Mann–Whitney *U* tests.

## Results

### Basic data

Eleven records were excluded because the questionnaire was not completed, 8 because they were duplicates of the same center, and 2 because not one question was answered. Seventy-one centers completed the online survey and were included in the analysis. The centers were located in Germany, Austria, Switzerland, and Luxemburg. In Germany, centers from 15 of 16 federal states participated. Of 71 participating centers, 40 were university/maximum care hospitals, 20 were standard care hospitals, and 11 were medical practice. The majority of the centers (*n* = 34; 47.9%) had more than 20 years of experience in pediatric gastroenterology. Twenty-two centers (31.0%) had an experience of 10–20 years, 10 centers (14.1%) had 5–10 years, and 5 centers (7.0%) had under 5 years of experience.

### Microbiome analysis

Twenty-two centers (31.0%) state to have performed or assessed microbiome analyses. Three of these centers stated that they perform analyses regularly (1/71; 1.4%) or frequently (2/71; 2.8%). Nineteen centers use external partners when performing microbiome analysis, and 4 centers are performing microbiome analyses in their own facility (48 answered “not applicable”).

Regarding the technique of microbiome analysis, 7 centers use 16S-RNA sequencing. Four centers stated that they use shotgun sequencing. Fourteen participants stated that the technology used was unknown (multiple choice question). Regarding the diagnostic value, 94.4% (67/71) of centers rated the value as low (1/71 “high,” 3/71 “unsure”).

### Fecal microbiota transfer

**Fig. 1:**
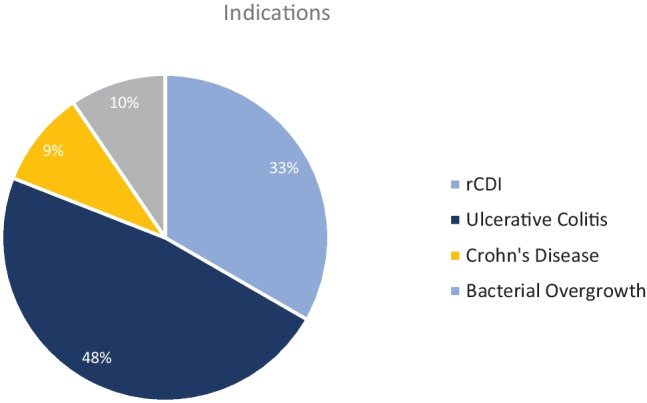
Question 9, indications for performing FMT; rCDI, recurrent Clostridioides difficile infection

11 centers (11/71; 15.5%) have performed FMT in their own facility. Two “FMT centers” stated that they have treated more than 5 patients (one more than 20). Concerning donor screening, 72.7% (8/11) of these centers stated that they use an in-house screening, and 27.3% (3/11) use an external screening program. Eight centers use fecal microbiota from donors related to the patient. Four centers stated that the donors were drawn from a stool bank (1 center uses both). Indications for performing FMT were assessed in a multiple-choice question. Ten centers performed FMT in patients with ulcerative colitis, 7 in patients with rCDI, 2 in patients with Crohn’s disease, and 2 in patients suffering from bacterial overgrowth (Fig. 1).

**Fig. 2:**
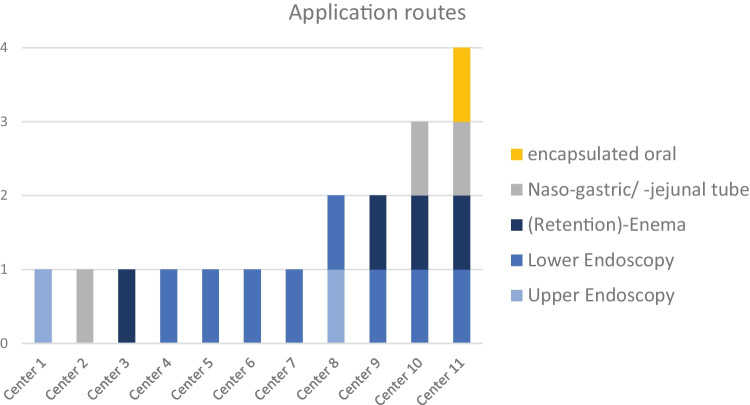
Question 10, application routes per FMT center

Regarding the application route, 8 centers reported that they perform FMT using lower endoscopy. Four centers have used retention enema, 2 have used upper endoscopy, and 3 used a nasojejunal tube. One center has experience with encapsulated FMT. Four centers use more than one application route (see Fig. 2). When performing FMT in IBD patients, 63.6% (7/11) of centers administer multiple doses, and all are using a weekly interval (2/11 administer only one dose; 2 FMT centers did not answer this question). In the assessment of adverse events, 4 out of 11 centers performing FMT (36.1%) reported adverse events, such as flatulence, diarrhea, and weight gain. In addition, two cases of transmission of viral infections (norovirus and adenovirus) were reported. The patient’s acceptance of FMT was rated as good by 27.3% of FMT centers (3/11). Nine centers (81.8%) stated that their patients’ acceptance was limited. One center chose both answers (“good” and “limited”). No center rated the acceptance as low. The clinician rating on the therapeutic effect of FMT was reported by 52 centers. Seven centers (13.5%) rated the effect as high and 17 (32.7%) as moderate. Fifteen centers (28.8%) rated the impact as questionable, and 8 centers (15.4%) rated it as low. Five centers (9.6%) gave the answer “neutral.” Regarding only FMT centers, 81.8% rated the therapeutic effect as high or moderate (3/11 and 6/11). The question on willingness to refer patients to other centers for FMT was answered with “yes” by 48 of all centers (67.6%). The same number of centers (*n* = 48; 67.6%) stated that they are willing to participate in clinical trials. 14 (19.7%) were not willing, and 8 (11.3%) were unsure. When asked about other therapeutic interventions, 63 centers reported to prescribe probiotics. 48 centers use antibiotics, and 28 centers use prebiotics to influence the intestinal microbiome. 16 centers use symbiotics, and 4 centers indicated “nutrition” as a free text response.

### Correlations

Concerning the basic characteristics, hospitals with maximum care have significantly more experience than smaller institutions (*p* = 0.003 compared to outpatient practice and *p* < 0.001 compared to standard care hospitals for the item “more than 20 years of experience”; unpaired *t*-test). Centers in maximum care hospitals were more likely to participate in clinical trials than smaller centers (83.3% vs 50%; *p* = 0.043; Fisher’s exact test comparing the categorial variables “medical facilities” and “willingness to participate in clinical trials”).

## Discussion

We conducted the first structured survey on the use of microbiome analysis and therapeutical fecal microbiota transfer in pediatric gastroenterology. With 71 participating centers, located all over the German-speaking community of pediatric gastroenterology, we evaluated representative data. Centers were predominantly located at university and maximum care hospitals and represented high-level specialization and interdisciplinarity.

### Analysis of microbiota composition

The majority of centers do not perform microbiome analysis in their clinical setting, and the diagnostic value is rated as low. This is an interesting finding as the scientific interest and private investment in this area is increasing. The lack of clearly defined reference values for the distribution and diversity of the microbiota, especially for the pediatric population, as well as the lack of knowledge on the meaning and the possibility of manipulation of potentially pathological microbiota patterns, limits the routine use of microbiota analysis. As only four centers perform analyses in their own facility, there is a clear lack of experience, and most centers rely on private provider. Costs must mostly be paid by the patient as no insurance covers the analysis of microbiota composition. Guidelines and protocols for performing clinically meaningful diagnostic microbiome analyses are needed urgently.

### Fecal microbiota transfer

Only 15% of pediatric centers have performed FMT. The selection and preparation of patients, donor screening, ethical and legal considerations, and equipment are demanding, time-consuming, and expensive. As the required infrastructure is complex, FMT should be performed in experienced maximum care facilities. Pediatric centers performing FMT mostly use individual in-house screening programs for potential donors. This means that the official guidelines and manuals, such as the consensus report from a multidisciplinary United European Gastroenterology (UEG) working group [11], and the joint position paper from the NASPGHAN and ESGPHAN on FMT in children with rCDI [12] are not regularly used. A consented donor screening program in pediatric FMT, according to the UEG consensus report, is essential for the safe performance of pediatric FMT. In addition, changes in the situation, such as the COVID-19 pandemic, require timely adaptation to ensure the safety of FMT [13]. The most frequently mentioned indication for pediatric FMT in our survey was ulcerative colitis, followed by recurrent CDI. Only one-third of pediatric FMT centers performed FMT in rCDI-patients. The UEG survey, interviewing FMT centers for adult patients, stated that all adult centers perform FMT in rCDI patients and that 57% of all procedures were performed with this indication [10]. This could be due to the much higher incidence rate of CDI in adult patients and the official guidelines mentioning FMT as the treatment of choice for rCDI [14]. Ulcerative colitis in adult patients is the leading experimental and investigational indication [10]. In assessing patients’ acceptance and potential therapeutic benefit of FMT, our survey showed mixed results. Overall, acceptance was mostly rated as limited by physicians. The possible therapeutic effect was rated mostly as moderate, neutral, or limited. This rather pessimistic view is in line with other surveys showing that physicians are often skeptical about FMT due to infectiological concerns [15]. These concerns are often based on a lack of information about safety and screening procedures [16]. This is supported by the fact that in our survey, FMT centers are much more optimistic in their view. Interestingly, the patients’ perception is also more optimistic. Ulcerative colitis patients see FMT as a promising therapeutic option [17], and patients after FMT highly recommend the procedure [15]. A higher recognition degree leads to a more positive attitude towards FMT, and popularization can promote the further development of FMT [17].

## Limitations and conclusion

More guidelines, national registries [18], and clinical trials on pediatric FMT are needed. Most centers in our survey were willing to transfer patients for FMT and were willing to participate in clinical studies. This is in compliance with other international surveys [19]. Consecutive to the FMT special interest group of ESPGHAN and NASPGHAN, a special interest group of the GPGE initiated the Microbiome Working Group in order to network and share knowledge with the aim of jointly conducting larger pediatric gastroenterology clinical trials in the future to improve patient care. Limitations of the survey are due to anonymity and sample size. Although FMT has been well established in the treatment of rCDI in adults, its role in pediatric gastroenterology is limited by the lack of dedicated centers, difficulties with donor recruitment, and complex regulatory rules and safety regulations [20]. Therefore, it is necessary to establish pediatric FMT centers with standardized procedures for patient selection, donor screening, application route, volume and frequency of use, and a structured register to assess feasibility and efficacy. These centers could also conduct larger clinical trials, e.g., for IBD patients, to improve care in pediatric gastroenterology.

## Supplementary Information

Below is the link to the electronic supplementary material.

Supplementary file1 (PDF 159 KB)

## Data Availability

The datasets generated during and/or analysed during the current study are available from the corresponding author on reasonable request.
