# Supplementary material for: Microbiome analysis and fecal microbiota transfer in pediatric gastroenterology — a structured online survey in German-speaking countries
Source: Int J Colorectal Dis. 2023 Mar 3;38(1):59. doi: 10.1007/s00384-023-04351-7 (PMC9982773; doi:10.1007/s00384-023-04351-7)
Supplement: Supplementary file 1 — Supplementary file1 (PDF 159 KB) [file 384_2023_4351_MOESM1_ESM.pdf]

UNIKLINIK KÖLN | Klinik und Poliklinik für Kinder- und Jugendmedizin

Direktor: Univ.-Prof. Dr. med. J. Dötsch

Uniklinik Köln · Klinik und Poliklinik für Kinder- und Jugendmedizin · Kerpener Str. 62 · 50937 Köln

Dr. med. Alexander Joachim
Facharzt für Kinder- und Jugendmedizin
Kindergastroenterologe und –hepatologe
Leitung Flüchtlingsambulanz

Telefon: +49 221 478-32636
Telefax: +49 221 478-32852
Zeichen:

Mikrobiomanalysen und Mikrobiomtransfer im deutschsprachigen Raum – Eine Umfrage bei pädiatrischen Gastroenterolog*innen

1. An welcher Einrichtung sind sie tätig
   - Uniklinikum / Maximalversorgende Klinik
   - Klinik der Regelversorgung
   - Praxis / MVZ

2. Seit wie vielen Jahren behandelt ihr Zentrum Kinder und Jugendliche mit gastroenterologischen Problemen?
   - < 5
   - 5-10
   - >10 - 20
   - >20

3. Wie häufig führen Sie Mikrobiomanalysen bei Ihren Patienten durch?
   - Nie
   - Vereinzelt
   - Regelmäßig
   - Häufig
   - Weiß nicht

4. Wo werden die Mikrobiom-Analysen durchgeführt?
   - In der eigenen Einrichtung
   - Extern
   - Nicht zutreffend

5. Welche Technik der Mikrobiomanalyse wird in der Regel genutzt?
- 16S-RNA Sequenzierung
- Shotgun Sequenzierung
- Andere:____
- Unbekannt
- Nicht zutreffend

6. Welchen Stellenwert haben Mikrobiomanalysen in ihrem diagnostischen Setting
- Niedrig
- Hoch
- Weiß nicht

7. Haben Sie in Ihrer Einrichtung bereits fäkal-Mikrobiomtransfers (FMT) durchgeführt?
- Ja
- Nein

8. Bei wie vielen Patienten Sie bereits FMTs durchgeführt?
- 0
- 1-5
- 6-20
- > 20

9. Bei welchen Indikationen wurde der FMT durchgeführt?
- (rezidivierende) CDI
- Colitis ulcerosa
- Morbus Crohn
- Reizdarmsyndrom
- Bakterielle Fehlbesiedelung
- Autismus Spektrum Störung
- Andere: ____
- Nicht zutreffend

10. In welcher Form wurden FMTs in Ihrem Zentrum bereits appliziert?
- Obere Endoskopie
- Untere Endoskopie
- (Retentions)-Einlauf
- Naso-gastrale/jejunale Sonde
- Verkapselt p.o.
- Andere: ____
- Nicht zutreffend

11. Wer war der/die Spender*in des FMTs?
    - Angehörige*r
    - Spender*in einer Stuhlbank der eigenen Einrichtung
    - Andere: ____
    - Nicht zutreffend

12. Welchem Screening wird der/die Spender*in vor dem FMT unterzogen?
    - Keinem
    - Screening-Programm anderer Einrichtungen
    - klinikeigenes Screening
    - hier können Sie Ihre Antwort präzisieren:________________________
    - Nicht zutreffend

13. Für CED: Wie ist in der Regel die Frequenz der Verabreichung?
    - Einmalig
    - Mehrfach
    - Nicht zutreffend

14. Bei mehrfacher Verabreichung, wie ist die Frequenz der Verabreichung:
    - Täglich
    - Einmal Wöchentlich
    - Andere:________________________
    - Nicht zutreffend

15. Haben Sie Komplikationen/Nebenwirkungen beobachtet?
    - Ja
    - Nein

    Wenn ja, können Sie die Komplikationen/Nebenwirkungen hier kurz schildern:
    ____
    ____

16. Wie beurteilen Sie die Akzeptanz durch die Patienten?
    - Sehr gut
    - Mit Einschränkungen
    - Schlecht

Sie haben hier die Möglichkeit zu einer Präzisierung:
____

17. Für CED: Wie schätzen Sie den möglichen therapeutischen Nutzen des FMT ein?

- hoch
- neutral/fraglich
- gering
- nicht zutreffend

Begründung, optional:____
____

18. Würden Sie ausgesuchte Patienten für einen FMT an andere Zentren überweisen?

- Ja
- Nein
- Unsicher

19. Würden Sie an klinischen Studien teilnehmen, die z.B. den Nutzen von FMT zur Behandlung von CED belegen sollen?

- Ja
- Nein
- Unsicher

20. Welche anderen Therapien nutzen Sie, um das Mikrobiom Ihrer Patienten zu beeinflussen?

- Probiotika
- Präbiotika
- Symbiotika
- Antibiotika
- Andere:____
- Keine
